# Perception of Drug Vendors and Pig and Poultry Farmers of Imerintsiatosika, in Madagascar, Toward Risks Related to Antibiotic Usage: A Q-Method Approach

**DOI:** 10.3389/fvets.2020.00490

**Published:** 2020-08-21

**Authors:** Chloé Bâtie, Daouda Kassie, Diary Ny Ranto Mamorisoa Randravatsilavo, Laurence Baril, Agnès Waret Szkuta, Flavie Luce Goutard

**Affiliations:** ^1^CIRAD BIOS, UMR ASTRE, Univ Montpellier, INRAE, Montpellier, France; ^2^Unité d'Épidémiologie et de Recherche Clinique, Institut Pasteur de Madagascar, Antananarivo, Madagascar; ^3^Département Vétérinaire, Faculté de Médecine, Université d'Antananarivo, Antananarivo, Madagascar; ^4^ENVT, IHAP, Université de Toulouse, INRA, Toulouse, France; ^5^Faculty of Veterinary Medicine, Kasetsart University, Bangkok, Thailand

**Keywords:** antibiotic resistance, communication, livestock, Madagascar, opinions, participatory epidemiology

## Abstract

Antimicrobial resistance is a One Health issue that must be tackled worldwide. In order to implement effective communication strategies in Madagascar, a better understanding must be gained of practices and perceptions related to antimicrobial use at the smallholder farm level. Our study used a semi-qualitative approach, called Q methodology, to identify patterns of opinion on antimicrobial use, or its alternatives, among pig and poultry smallholders and drug vendors in the commune of Imerintsiatosika, in Madagascar. Twenty-nine breeders and 23 drug vendors were asked to rank, respectively, 38 and 45 statements, produced from semi-structured interviews and secondary data, through a 7 grade scale from −3 (totally disagree) to +3 (totally agree) about antimicrobial use, related risks and alternatives. The interview ended with a discussion around extreme statements. The *Q-sortings* were analyzed by factor analysis and Principal Component Analysis. Regarding antimicrobial use, antimicrobial resistance and alternatives, the breeders and drug vendors were divided according to three discourses: “A: confidence in antibiotics” (respectively, 13 and 6 individuals), “B: belief in alternatives” (7 and 7 individuals), and “C: moderate approach to antibiotic use” (6 and 6 individuals), explaining, respectively, 57 and 60% of total variance. Group A was associated with the use of antibiotics as a preventive measure, poor knowledge of resistance and low trust in alternatives. Group B considered the preventive use of antibiotics to be a major problem for antimicrobial resistance and believed that alternatives, such as vaccines, were useful preventive methods. Group C seemed to have a hazy opinion. The presence of three main points of view offers the possibility to adapt awareness messages. Group B might also be used as a showcase to reduce the amounts of antibiotics used by the two other groups. This study revealed different practices and risk perceptions related to antimicrobial use that must be better characterized and accurately quantified.

## Introduction

Antimicrobial resistance (AMR) is currently one of the main public health threats worldwide and the misuse or overuse of antibiotics (AB) in human and veterinary medicine is one of its main drivers (1). One of the general recommendations of the World Health Organization (WHO), World Organization for Animal Health (OIE), and Food and Agricultural Organization (FAO) to tackle the problem is to improve awareness and understanding of AMR among the public and professionals.

As end-users of AB and main providers, breeders play a key role, in antimicrobial usage (AMU), toward reduction strategies and the prevention of spreading resistance (2). Veterinarians are also important actors in the fight against AMR. They act as consultants in farm management, oversee treatment, and write the prescriptions required to buy drugs in most countries worldwide. They are considered as the most legitimate persons to inform breeders on usage, risks, and alternatives to AB (3, 4). The relationship between veterinarians and farmers is also critical. Visschers et al. (5) show that, in Europe, breeders who systematically call the veterinarian use smaller amounts of AB.

Since 2014, studies have been conducted in Europe to explore the perception of breeders and veterinarians toward AMU, AMR, alternative treatments and policy measures (2, 5–7). This has enabled an evaluation of people's understanding of the issue, the identification of their motivations and an alleviation of certain barriers to change. Hence, their perceptions can be a base from which to elaborate more effective communicative strategies. However, despite the fact that low and middle income countries use large amounts of AB with, lately, a significant increase in their consumption (8), few studies have been completed on farmers' perceptions.

Madagascar is among the 10 poorest countries in the world (9). With its extreme natural wealth and great geographical diversity, agriculture is among the major economic sectors of the country. Indeed, 78% of the population live in rural areas and 60% breed animals as a source of income (10). In most families, livestock is a capital that can be used in the case of financial difficulties or for self-consumption (11). Poultry is the most commonly farmed livestock, with almost 35 million animals, followed by cattle (10 million) and pigs (1.5 million) (11). Although some intensive commercial farms do exist, most production are backyard (free-range animals, no care provided) or semi-intensive (small contained headcount, minimal care) farms (12). AMR is of public concern, with resistance reported in humans for *Staphylococcus aureus, Enterococcus* spp., *Pseudomonas aeruginosa, Acinetobacter baumannii*, and *Enterobacteriacae* spp. The latest, including extended-spectrum β-Lactamase and carbapenemase-producing Enterobacteriaceae (ESBLE and CPE), was described by the Indian Ocean Commission (IOC) as one of the main human and animal threats (13). In Madagascar, due to the lack of sanitation, the close contact between humans and animals, and the difficulties to access medical care, resistance will become one of the highest burdens over the coming decades. Little information has been published on AMU or AMR in the livestock sector in Madagascar (13). Crépieux (12) suggests that there is poor knowledge of AB and a high percentage of self-medication. In a recent study, the prevalence of ESBLE in pigs, cows, and poultry was higher than 65% and reached 86.7% in swine (14). The situation remains unclear, and more data must be collected on knowledge and perceptions within the animal sector.

The qualitative approach, including participatory epidemiology, is an interesting method with which to establish an initial assessment of a problem. It is a *bottom-up* method (15) based on the active participation of individuals in defining their own solutions tailored to their issues (16). By identifying the major characteristics of a problem, it can be used as a baseline for the development of further studies. Usually cheaper than conventional studies, it allows the collection of information that is sometimes difficult to access (17). The most frequently used methods are informal interviews, visualization, ranking and scoring tools (18).

To evaluate the impact of AMR and develop alternatives to AB in Madagascar, our first step consisted in studying the perceptions of livestock professionals in the region of Itasy, including breeders and drug vendors, toward AMU and AMR. We used a semi-qualitative method called Q-methodology to identify patterns of opinions (19) and to understand AMU practices, the perception of related risks and attitudes toward alternatives. This method helped us to determine common and distinct opinions within our study population. As decision-making processes can be influenced by socio-demographic factors, we also studied their impact on perception.

## Materials and Methods

### Study Zone and Population

**Figure 1:**
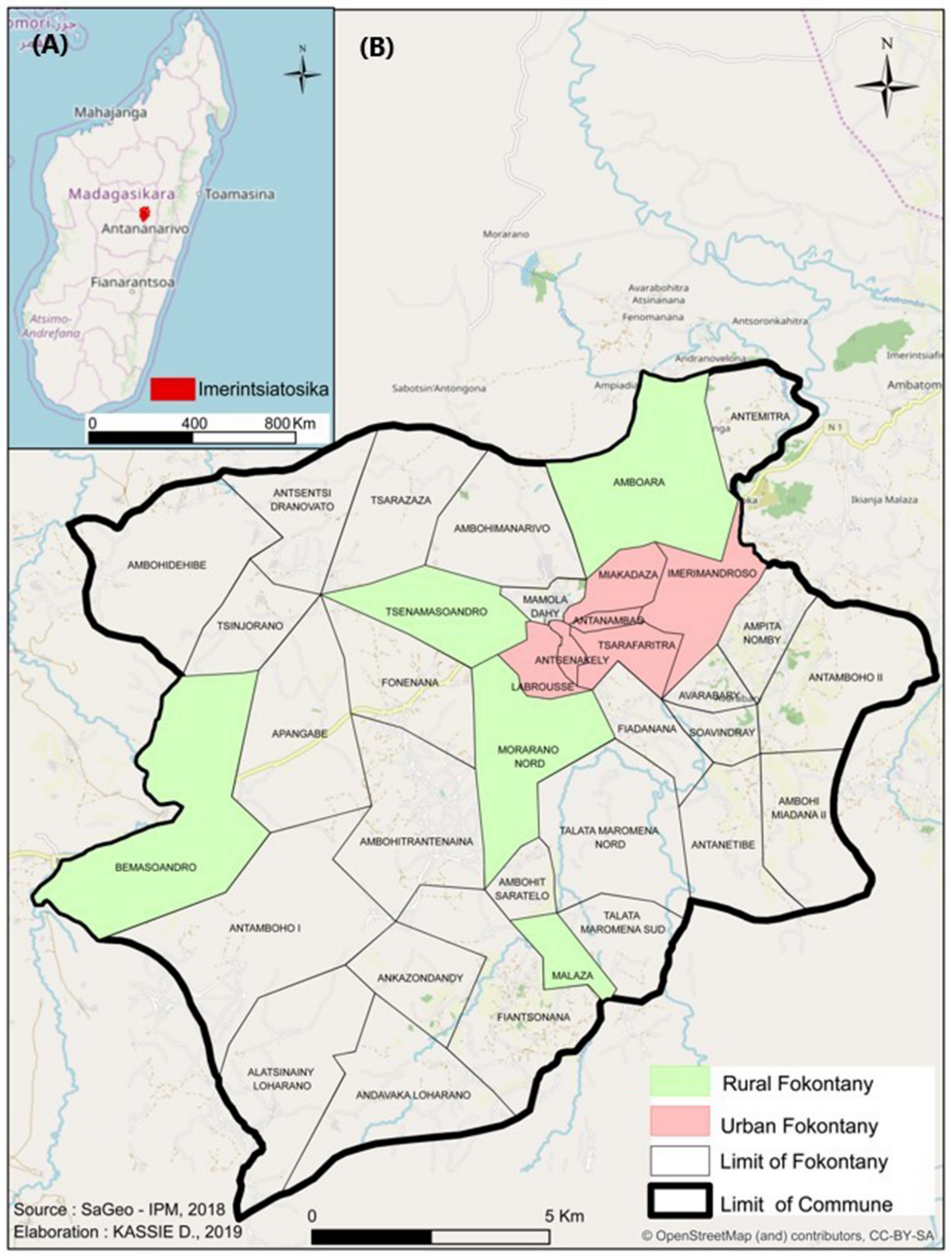
Map of the study area in Madagascar **(A)** and Imerintsiatosika **(B)**. Red: urban studied fokontany; green: rural studied fokontany.

This study was conducted in the commune of Imerintsiatosika, 30 km from Antananarivo (the capital city) in the region of Itasy, from April to May 2018. The high density of pig and poultry farms and easy accessibility were the main criteria used in the selection of this zone. The city is divided into 36 fokontany (the smallest administrative unit in Madagascar) and subdivided into urban or rural fokontany. An urban fokontany is defined by a certain density of urban construction and then was confirmed by the respondents during the survey (name of the fokontany and rural/urban fokontany). Our study zone included six urban (Antanambao, Antsenakely, Labrousse, Imerimandrose, Miakadaza, and Tsarafaritra) and five rural (Amboara, Bemasoandro, Malaza, Morano Nord, and Tsenamasoandro) fokontany (Figure 1), also chosen for their accessibility. Our first population was poultry and/or pig breeders, including family smallholdings (between 1 and 10 pigs and up to 100 poultry), semi-intensive farms (between 10 and 100 pigs and up to 500 poultry), and intensive farms (more than 100 pigs and up to 2,000 poultry) of the commune. Because most of the statements are based on the assumption that respondents have a minimum knowledge about AB, breeders who do not administer AB to their animals were excluded. Our second population was drug vendors including veterinarians, technicians, and other salesmen working with the breeders of Imerintsiatosika. Following interviews, professionals from Antananarivo and Ambatomirahavavy (between Imerintsiatosika and Antananarivo) working with breeders in Imerintsiatosika were also included.

### Q-Methodology

Q-methodology was used to explore the perception of breeders and drug vendors toward AMU. This semi-qualitative method studies the subjectivity of individuals regarding a complex and sensitive subject. Its main objective is to identify groups of individuals sharing the same point of view and to determine common and distinct opinions on a same subject by means of correlation (20). The precise methodology is described by Exel and Graaf (19).

Q methodology follows five steps: generation of the *concourse* (list of statements), construction of a set of statements (the *Q-set*), selection of the respondents (the *P-set*), ranking of the *Q-set* (the *Q-sorting*) and finally the analysis and interpretation of the factors (19).

#### Generation of the Concourse

The *concourse* is the raw material of the method, defined as “the flow of communicability surrounding any topics” (21). It consists of a list between 200 and 300 statements representatives of all the opinions and ideas about the subject (22).

In our study, the *concourse* was conceived firstly through a literature review of documents related to AMR and animal production (worldwide and in Madagascar), and then by implementing tools from participatory epidemiology (PE), in particular, direct observations and semi-structured interviews (SSI) of key informants (18). The literature review allowed us also to determine relevant information that needed to be include in the guide of interviews. The SSI were based on a check list including topics such as antibiotic use, antibiotic advice, relationships between farmers, veterinarians, and drug vendors, risk of antibiotic use for animals and humans, and knowledge about alternatives to antibiotics.

In order to get the maximum amount of information to help us to create the Q-set, we conducted the interviews with people working in different institutions as well as some breeders and sellers. As it is difficult to have the representativeness of all the types of actors intervening in the antibiotics sector, the interviews were therefore contained with some people with whom we were able to establish contacts and who are also key actors in Madagascar. The SSI were conducted in Antananarivo with 4 persons [2 employees from the direction of veterinary services, 1 from the National Research Center applied to rural development (FOFIFA) and 1 veterinary student] and, in Imerintsiatosika, with 8 persons (1 private veterinarian mandated by the veterinary services in charge of the commune, 3 drug vendors, and 4 breeders). At the end, the formulation of the statements include in the *concourse* was done from the literature review and the SSI of key informants.

#### Construction of the Q-Set

The Q-set is a list of statements built from the *concourse*. We first organized the information collected from the literature review and SSI into a list of statements around three main topics (use/advice on antibiotics, risk of using antibiotics, use of alternatives). The organization of the statements around the three main topics was done following an inductive approach. We started from a raw list of statements that we organized according to their similarities. The selection of the statements was done according to the relevance of the statements for the objectives of the study by the research team. Then, the statements were separated among our two study populations (drug vendors and breeders) and organized into sub-topics. To reduce the *concourse* to a manageable *Q-set* (between 30 and 60 statements), we removed statements with similar assertions. These were reviewed by three different researchers, who were familiar with the subject, to evaluate their relevance and understanding. They were then translated into Malagasy by the research assistant and reviewed by one veterinary student from Madagascar. We printed the statements on separate cards, which were randomly assigned a number. Finally, 2 drug vendors and 4 breeders were used to pilot the study protocol.

#### Selection of the P-Set

The *P-set* is the set of individuals interviewed, usually less than the number of statements. The goal is not to be representative of the population but to obtain a wide array of existing opinions (19). They are not chosen randomly but according to some socio-demographic characteristics considered to be relevant in the subject. Our respondents were identified with the help of the veterinarian in charge of food safety in the area, of his assistant and through a snowball sampling. We planned to include 30 breeders according to the species present in their farms (pigs, poultry, or both), their location (urban or rural) and their type of production (familial, semi-intensive, or intensive), and 30 drug vendors according to their type of activity (veterinarians, technicians, and sales representatives) and their link with the veterinarian in charge of this commune (independent, working with him, working for a company).

#### Statement Sorting and Ranking

The face-to-face interview was undertaken by the principal investigator and the research assistant, both of whom were trained in PE methodology. Throughout the presentation of the study, respondents were informed about the objective, the duration (around 1 h) and were provided with instructions to complete the tasks. Before the application of the method, a questionnaire on socio-demographic characteristics was completed by the respondent. For breeders, topics concerned age, gender, years of experience, education level (elementary school, middle school, high school, university), species (poultry, pigs or mixt), type (smallholding, semi-intensive, intensive), working status in the farms (owner or employee), and location (urban or rural fokontany). For drug vendors the topics were age, gender, years of experiences, jobs (veterinarians, technicians, or other salesmen), relationship with the veterinarian of the commune and training (presence or absence).

**Figure 2:**
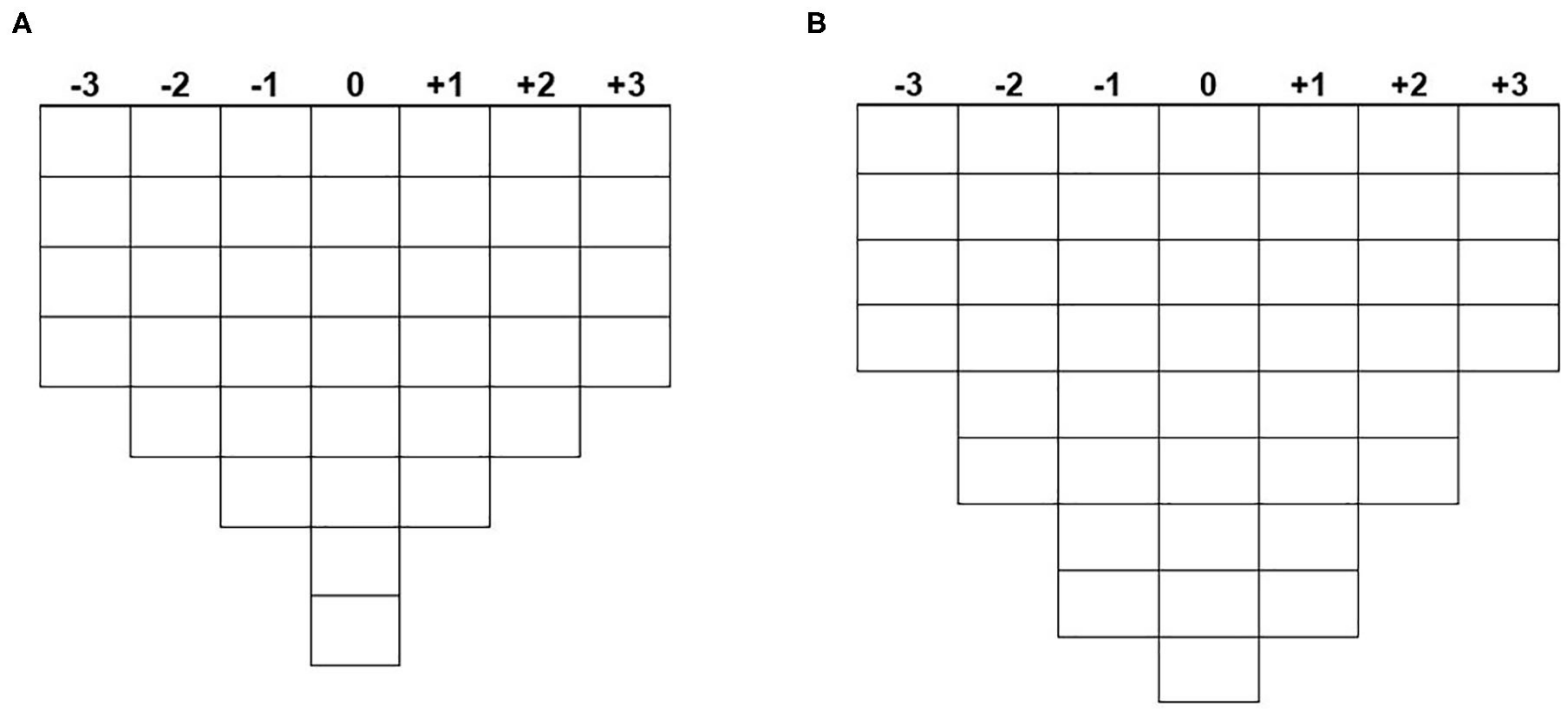
Q-sorting. Breeders: 38 statements **(A)**; drug vendors: 45 statements **(B)**.

The respondents were then asked to rank the *Q-set* (meaning all of the cards) according to certain rules called *conditions of instruction* and to their own point of view. The first step was to place all the statements into three piles: “agree,” “neutral,” “disagree.” Then they were asked to place each statement in a seven grade grid from +3: “totally agree” to −3 “totally disagree,” 0 corresponding to “neutral,” following a forced distribution (Figure 2). The cards were read out loud for people who were not able to read by themselves. During the interviews, they were free to move the cards as they wanted until they finally agreed with the position of all cards. Finally, a discussion was held on the extreme statements (+3 or −3) and sometimes certain other specific statements. At the end, we obtained a *Q-sort* for each participant. It is the result from the ranking of the *Q-set* by the respondent following the *conditions of instruction* (each statement have a grade between −3 and +3 allocated) and that represents an individual subjective pattern.

### Data Analysis

#### Quantitative Analysis

Data analyses were run using R 3.4.1. software with “FactoMineR” and “qmethod” packages. The analytical process is described by Zabala (23). Data are presented in a matrix with statements in rows and *Q-sorts* in columns. First the inter-correlation matrix is calculated, it represents the correlation between each *Q-sorts*. Then, this matrix is reduced, and factors are extracted using Principal Component Analysis (PCA). Adversely to a classic PCA, *Q-sorts* are the variables and disposed in columns in the matrix. The number of principal components chosen for further analysis are based on the following criteria: the eigenvalue of the component should be greater than one, the total variance explained >40%, more than two *Q-sorts* should be loaded by factor and the factors should make sense once the analyses is completed.

The selected factors are then rotated with the Varimax mode to maximize the association between the variables, the *Q-sort* and the factors, and to obtain a clearer structure. To each *Q-sort* a factor loading is calculated and represent the relation between *Q-sorts* and factors. Then, the *Q-sorts* which define a factor are flagged. It belongs to a factor when it follows the two equations: $l>1,96\sqrt{N}$ and $lj>\;\overset{f}{\underset{i=1}{\sum }}l^{2}i-l^{2}j$ where *l* is the factor loading, *N* the number of statements and *j* the considered factor (*p* < 0.05) (24). *Q-sorts* which do not respect this or that load to more than one factor are called confounding. For the next steps, only flagged *Q-sorts* are used in further calculations. Then the *z*-score is calculated. It represents the relationship between a statement and a factor. The factor score is the normalized weighted average statements scores (*z*-scores). It permits to have factors with different perspectives (a point of view or opinion). At the end, we obtain a factor score for each statement of each factor, that represent the score that an ideal respondent which loaded 100% with the factors will respond. The interpretation of the factors is based on these scores. When, for a statement the *z*-scores are statistically different between factors (more than a threshold based on the *SE* of differences between two factors multiplied by 1.96 for *p* < 0.05), it is called “distinguishing statement” (23). If there are no statistical differences between any pair of factors, it is considered as a “consensus statement” (the same opinion is shared by all factors).

#### Socio-Demographic Characteristic Analysis

To identify variables that could describe respondents within a same discourse, the socio-demographic characteristics from the questionnaire were analyzed with a Kruskal-Wallis test for non-parametric data on the R software.

#### Qualitative Analyses

Recorded interviews were transcribed and then translated into French or directly transcribed into French on WORD Microsoft Office 365 software version 2016. Where a recording device was not used, interviews were recorded in writing. The different factors were analyzed and interpreted using the ABC model of attitudes for sociological sciences (25). This model helps to study attitudes by describing them through three components: Affective, Behavioral, and Cognitive. Attitudes toward antibiotics were decomposed into three components: an affective component (feelings of breeders and drug vendors toward antibiotics, alternatives and relationships between the two categories of population), a behavioral component related to action (the AMU, advice and alternative usages) and a cognitive component (belief and knowledge about AMU, risks and alternatives).

## Results

### The Q-Set

Two hundred and forty-five statements were formulated based on the literature review and the SSI. After categorization, reviewing and removal of duplicates, 55 statements for breeders and 47 for drug vendors were retained and translated into Malagasy. After the two field test sessions, the final *Q-set* was composed of 38 statements for breeders and 45 for drug vendors.

### Breeders

#### Presentation of the P-Set

Thirty-one interviews were conducted in the area of Imerintsiatosika. Two farmers did not meet the inclusion criteria (no use of antibiotics and over 2,000 poultry). During data analysis, three *Q-sorts* (so three sets of answers from respondents) were found to be confounding and were removed from the analysis.

Out of the 26 respondents, the majority were men (17/26) and younger than 40 years of age (18/26). Participants were mainly educated (19/26 reached at least high school). Most breeders owned their farm (21/26), had at least one pig (21/26) and worked according to a semi-intensive model (16/26). The number of farms located in rural areas was quite similar to the number in urban areas (46 and 54%, respectively). Finally, the average number of years of experience was 10.38. Details of the socio-demographic characteristics are presented in Table 1.

**Table 1:** Socio-demographic variables and result of Kruskal-Wallis test for the whole population of breeders (a), people belonging to F1 (b), F2 (c), and F3 (d).

**Variables**		**(a)**	**(b)**		**(c)**		**(d)**	
		**Population**	**F1**	***P-*value**	**F2**	***P-*value**	**F3**	***P-*value**
Number		26	13		7		6	
Gender	Woman	9	6	0.53	1	0.39	2	0.08
	Man	17	7		6		4	
Age	≤ 40	18	11	0.02^*^	4	0.059	3	0.33
	>40	8	2		3		3	
Experience	(years)	10.38	10.7	0.48	6.8	0.83	13.8	0.21
Education	Elementary sch.	4	1	0.07	0	0.009^**^	3	0.13
	Middle sch.	3	1		1		1	
	High sch.	10	7		1		2	
	University	9	4		5		0	
Status	Owner	21	10	0.67	7	0.008^**^	4	0.74
	Employee	5	3		0		2	
Specie	Pork	10	6	0.10	3	0.19	1	0.76
	Poultry	5	1		3		1	
	Mixt	11	6		1		4	
Type	Familial	5	3	0.89	2	0.49	0	0.85
	Semi-intensive	16	8		3		5	
	Intensive	5	2		2		1	
Farm localization	Urban	14	8	0.71	2	0.85	4	0.55
	Rural	12	5		5		2	

* *p < 0.05*;

** *p < 0.01*.

#### Factor Analysis

Following the PCA, eight factors showed an eigenvalue above 1 and explained more than 50% of the total variance. After rotation, only three factors were extracted. These accounted for 57% of the total variance, loaded more than two *Q-sorts* and were the most meaningful. General factor characteristics, *Q-sorts* factor loadings and flagged *Q-sorts* (*Q-sorts* belonging to a factor) are presented in Supplementary Material.

The *Q-set, z*-scores and *Q-sort* values for each factor are presented in Table 2. The analysis of the factors was based on statements with extreme values (−3, −2 and +2, +3), the distinguishing statements and the content analysis of each individual interview. Following the analysis, the three discourses were named A “trust in antibiotics,” B “belief in alternatives,” and C “moderate use of antibiotics.” The Kruskal Wallis test performed between the three discourses showed that the education level was statistically different between the three groups (*p* < 0.01) with group C having a lower level of education (Supplementary Material).

**Table 2:** *Q-set*, rank and z-scores for the P-set breeders.

		**Factor**					
		**F1**		**F2**		**F3**	
**N^**°**^**	**Statements**	**Rank**	***z*-score**	**Rank**	***z*-score**	**Rank**	***z*-score**
1	When a neighboring farm has sick animals, it is necessary to treat immediately with antibiotics	1	0.79	−1^†^	−0.41	1	0.98
2	Breeders can treat an animal with any dose of antibiotics	−3	−1.93	−3	−1.75	−3	−1.37
3	If breeders use too much antibiotics on animals, we will not be able to treat some human diseases	0	0.08	1^†^	0.63	0	−0.06
4	**We must always ask advice to drug vendors before using antibiotics**	**3**	1.41	**2**	0.96	**2**	1.08
*5*	**We must always respect the withdrawal time of the antibiotics before slaughtering an animal**	**3**	1.27	**2**	1.01	**2**	0.98
6	Antibiotics use on animals can be dangerous for human health	−2	−1.09	0^†^	−0.09	−1	−0.73
7	The respect of the withdrawal time of the antibiotics protect consumers health	2	0.94	1	0.82	1^†^	0.28
8	*If breeders always use the same antibiotic to treat their animals, the antibiotic will not be efficient anymore in their farms*	*0*	0.41	*3*	1.30	*0*	−0.40
9	Everybody can enter in farms	−3	−1.67	−3	−1.85	−3	−2.26
10	Preventing diseases by antibiotics can lead to inefficient antibiotics	0	−0.18	2^†^	1.18	0	−0.15
11	*Vaccination can reduce antibiotics use in a farm*	*1*	0.76	*3*	1.29	*−1*	−0.67
12	**Antibiotics use in farms are always efficient**	**−1**	−0.56	**−1**	−0.55	**−1**	−0.51
13	Giving antibiotics is the cheaper way to prevent disease	1^†^	0.52	−2	−1.11	−2	−0.81
14	*We can stop antibiotics treatment when the animal is getting better*	*0*	−0.17	*−1*	−0.78	*1*	0.62
15	*Other methods exist to prevent diseases*	*1*	0.59	*2*	1.24	*−1*	−0.51
16	To prevent diseases, breeders should always use antibiotics on imported chicken	2^†^	0.89	0	−0.23	0	0.18
17	*Breeders use antibiotics to accelerate animal growth*	*2*	0.84	*−3*	−1.67	*−2*	−0.82
18	**Requiring prescription to buy antibiotics represent a loss of time and money**	**−2**	−0.84	**−1**	−0.51	**−2**	−1.00
19	Breeders must follow treatments advice from other breeders	−2	−0.61	−2	−0.81	0^†^	−0.02
20	The veterinarian or the technician always explain to the breeders which antibiotic he is using when he treats an animal	1	0.42	1	0.50	2^†^	1.04
21	Respecting the withdrawal time is a waste of money so breeders don't respect it each time	−1	−0.50	−1	−0.55	0^†^	0.20
22	**If prescription by veterinarians was mandatory to buy drugs, breeders would use less antibiotics**	**0**	0.04	**0**	−0.12	**0**	−0.27
23	Drug vendors should inform breeders about risks related to antibiotics use	0	0.01	0	0.35	2^†^	1.02
24	*We can breed without using antibiotics*	*−3*	−1.84	*0*	0.00	*−1*	−0.76
25	Expensive antibiotics are more efficient	−1	−0.40	0	−0.24	1^†^	0.54
26	Antibiotics can be used to treat any kind of infections	−1	−0.40	−1	−0.67	3^†^	1.26
27	Whenever an animal is sick, breeders can always use the same antibiotics	−1	−0.45	−2	−1.30	−2	−0.87
28	Breeders treat themselves with antibiotics because veterinarians and technicians are often busy	0	0.19	1^†^	0.49	−1	−0.51
29	If breeders separate sick animals from healthy one, we can prevent spreading disease	2	1.08	3	1.54	3	1.48
30	**Breeders should often clean the farm to have less disease**	**3**	1.89	**3**	1.87	**3**	1.82
31	The place where we buy antibiotics doesn't matter	−2	−1.21	−2	−0.99	0^†^	0.22
32	Breeders use antibiotics only when animals are sick	−2^†^	−1.48	2	1.16	1	0.86
33	**Antibiotics residues can be found in soil and rivers**	**0**	0.18	**0**	0.37	**1**	0.35
34	*When an animal is sick, breeders must always call the veterinarian*	*3*	1.77	*1*	0.49	*2*	1.22
35	*The veterinarian is expensive that's why breeders don't call him every time*	*1*	0.66	*0*	0.14	*−3*	−1.12
36	If the antibiotic is expensive, we need to reduce dosage	−3	−1.57	−3	−1.55	−3	−2.06
37	Eating meat (pigs or poultry) raise without antibiotics is better to human health	−1	−0.40	1^†^	0.64	−2	−0.83
38	*Using antibiotics is safe for animals*	*2*	0.95	*−2*	−0.79	*3*	1.62

† *Statement distinguishing factor from the rest. In bold: consensus statements; in italic: distinguish statements*.

#### Consensus Statements

Seven statements were consensus statements. When asked about the need for a prescription when buying AB, all the breeders agreed that it was not a waste of money or time (statement 18): the factor scores given by the breeders was −2 or −1 with no statistical difference within the two values (stat. 18: −2, −1). Indeed, they all believed advice from vendors to be necessary when using drugs (stat. 4: +3, +2), in particular regarding dosage (an overdose would lead to severe illness or mortality and an underdose would be ineffective). They did not have a strong opinion regarding the fact that prescriptions decrease the amount of AB used by farmers (stat. 22: 0), suggesting that they did not perceive the need for a prescription as a barrier to AB usage. They considered that cleaning the farm is a good way to reduce disease and consequently AB usage (stat. 30: +3), as “*property is a source of health”* E28/R25 (corresponding to answer 25 of breeder number 28). They did not believe that AB are always efficient (stat. 12: −1) saying that it depended on the disease and administration method. Concerning residues, they all believed that is was mandatory to respect the withdrawal time (stat. 5: +3, +2), but they had no understanding of residues in the environment (stat. 33: 0, +1).

#### Discourse 1A “Trust in Antibiotics”

This point of view was shared by 13 breeders and represents 23% of total variance. This discourse was influenced by the age of respondents, with statistically more people under the age of 40 (11 respondents were under 40 and 2 were over 40) (*p* = 0.02) (Table 1). This group was characterized by a positive opinion of AB and weak AMU practices, bad practices related to AMU misuse and overuse of AB. They strongly disagreed with the possibility of farming animals without using AB (stat. 24: −3), considering this to be unprofessional. AB were not used only when an animal was sick (stat. 32: −2) but also to prevent diseases and stress, particularly in imported chickens that were considered to be less resistant than local breeds (stat. 16: +2) “*I take an example, if today it's really warm and that the next day it's cold, we must use AB [.] and also when the animal moves on to a finisher feed after the growth feed, meaning that there is a dietary transition, we should use AB as it helps avoid animal stress. It's the instructor who taught us to use AB as prevention [.] and it works!"* E27/R13. AB were used as growth promoters (stat. 17: +2). This was consistent with the rest of their discourse concerning the safety of these products. As in group 1C, AB were considered to be safe for animals (stat. 38: +2) as well as for humans (stat 6: −2). But the prescription must be respected (made by the manufacturer that is a trusted professional) and AB should be bought in a specific place (stat. 31: −2), as agreed in group 1B. In accordance with this, it was considered important for them to call the vet when an animal is sick (stat. 34: +3), as they are trained, and therefore the best person to advise them. Meanwhile, they also treated by themselves when they were familiar with the disease, as breeders also have effective knowledge and experience, and the veterinarian is sometimes busy or considered to be too expensive. Finally, they did not have a clear opinion regarding the existence of other disease prevention methods (stat. 15: +1) or of the ability of vaccination to reduce AMU (stat. 11: +1). Thus, they did not consider these alternatives as a good means to reduce AB.

#### Discourse 1B “Belief in Alternatives”

The second discourse included seven breeders and explained 18% of total variance. This discourse was statistically influenced by two variables: farm owners (*p* < 0.01) and more educated people (5 university, 1 high school, 1 middle school, 0 elementary school, *p* < 0.01) (Table 1). This discourse was characterized by a more critical opinion of excessive AMU. Unlike the other groups, using AB was not considered to be safe for animals (stat. 38: −2). Excessive use leads to AB resistance and, therefore, they cannot be used as growth promoters (stat. 17: −3) or for disease prevention (stat. 10: +2) “*AB are not made to prevent diseases but to treat them […] when a disease appears, no antibiotics will be efficient; and it becomes, it becomes, microbes become resistant to drugs”* E31/R30. This was coherent with the rest of their discourse even if it was not statistically specific to this group: they used AB, but only as a treatment (stat. 32: +2). Moreover, the consistent use of the same AB was linked to a loss of effectiveness (stat. 8: +3). This group also trusted alternatives (stat. 15: +2). Vaccination was considered as the best strategy to prevent disease and reduce AMU (stat. 11: +3).

#### Discourse 1C “Moderate Approach to Antibiotic Use”

This group included six breeders, representing 14% of total variance. It was an heterogenous group as there were no significant differences between variables (Table 1). Contrary to the other groups, which differed in their strong opinion about AMU (one group is really in favor of AMU whereas the other promote a more prudent use), they did not have a clear and specific point of view regarding AMU (stat. 24: −1), AMR (stat. 8: 0) and alternatives (stat. 15: −1). For factor 1A, they believed that AB was safe for animals (stat. 38: +3) and for humans as it protects meat from contamination by bacteria (stat. 37: −2). But they seemed to have a moderate use of antibiotics because they were never used as growth promoters (stat. 17: −2). They also thought that AB could be used to treat any kind of infection (stat. 26: +2).

By contrast to the two other factors, the relationship between breeders and veterinarians was more present. They trusted advice given by veterinarians and technicians who always explain the treatment (stat. 20, +2). So, they need to call them when an animal is sick (stat. 34, +3) regardless of economic considerations (stat. 35, −3). They also considered veterinarians and technicians to be the best persons to explain risks to them (stat. 23, +2).

### Drug Vendors

#### Presentation of P-Set

Twenty-four people were interviewed in Imerintsiatosika, Ambatomirahava, and Antananarivo. One person was excluded because of an incoherent discourse. Four *Q-sorts* were confounding and excluded from further analysis. This population was mainly composed of men (12/19), of < 40 years old (14/19). Technicians represented more than half of the total population (10/19) followed by sales reps (7/19) and veterinarians (2/19). Twenty-six-point three percent worked for chicken production company that was authorized to sell drugs. Most of the drug vendors received training (13/19) and the average number of years of experience was 12.9. Details on socio-demographic characteristics are presented in Table 3.

**Table 3:** Socio-demographic variables and result of Kruskal-Wallis test for the whole population of drug vendors (a), people belonging to F1 (b), F2 (c), and F3 (d).

**Variables**		**(a)**	**(b)**		**(c)**		**(d)**	
		**Population**	**F1**	***P-*value**	**F2**	***P-*value**	**F3**	***P-*value**
Number		19	6		7		6	
Gender	Woman	7	2	0.24	3	0.83	2	0.97
	Man	12	4		4		4	
Age	≤ 40	14	4	0.75	6	0.10	4	0.68
	>40	5	2		1		2	
Experience	(Years)	12.9	12	0.50	7.7	0.73	20.17	0.39
Job	Other salesman	7	3	0.23	1	0.06	3	0.11
	Technician	10	3		4		3	
	Veterinarian	2	0		2		0	
Relation with	Company	4	0	0.38	3	0.14	1	0.58
	Independant	8	3		2		3	
	Veterinarian	7	3		2		2	
Training	Yes	13	2	0.01^*^	5	0.46	6	0.33
	No	6	4		2		0	

* *p < 0.05*.

#### Factor Analysis

After the PCA, six components had an eigenvalue of more than 1 and explained more than 50% of the total variance. Three main factors were retained, accounting for 60% of the total variance (Supplementary Material). The main ideas of each discourse, like those of breeders, led to the same group name and flagged *Q-sorts* which are presented in Supplementary Material. Analysis and interpretation of discourses was based on Table 4.

**Table 4:** *Q-set*, rank and z-scores for the P-set drug vendors.

		**Factor**					
		**F1**		**F2**		**F3**	
**N^**°**^**	**Statements**	**Rank**	***z*-Score**	**Rank**	***z*-Score**	**Rank**	***z*-score**
1	*Antibiotics to prevent disease are essential to the functioning of the farm*	*2*	1.30	*−2*	−0.94	*−1*	−0.31
2	Veterinarians or technicians should move to the farm when animals are sick	3	1.51	1	0.79	2	1.14
3	*Wholesalers have enough choice of antibiotics*	*1*	0.40	*−1*	−0.68	*2*	1.38
4	Eating meat (pigs or poultry) raise without antibiotics is better for human health	1^†^	0.95	0	0.02	−1	−0.47
5	It is necessary to inform breeders about dosage and length of treatment	2^†^	1.36	0	0.29	1	0.35
6	In first intention, we must use the most efficient antibiotics	1	0.31	1	0.51	0	−0.04
7	Some drug vendors sell fraudulent drugs	−1	−0.49	0	−0.14	2^†^	1.14
8	*There is no risk to use antibiotics If we respect the utilization advice*	*1*	1.06	*0*	0.24	*3*	1.65
9	Pigs or poultry can be raised without using antibiotics	−2	−1.44	1^†^	0.55	−1	−0.91
10	If an antibiotic is no longer efficient on an animal, it will also not be efficient on the other animals	−2	−0.71	−1	−0.86	−3^†^	−1.47
11	Dosage and length of treatment must always be written for the breeders	3^†^	1.59	1	0.65	1	0.49
12	If we always give the same antibiotics to breeders, it will become inefficient	1	0.31	1	0.53	−1^†^	−0.26
13	We must always ask questions on clinical signs or examine the animals before using an antibiotic	3	1.67	2^†^	0.80	3	1.87
14	Antibiotics use on animals is harmful for human health	−1	−0.39	0^†^	0.33	−1	−0.85
15	It is useless to talk about antibiotic's risks to breeders	0	−0.37	−2^†^	−1.40	0	−0.13
16	*Using antibiotics as preventive methods can lead them to become inefficient*	*−2*	−0.82	*1*	0.50	*0*	−0.21
17	Antibiotic choice depends of animal's symptoms	2	1.24	2	1.25	3^†^	1.86
18	If we misuse or overuse antibiotics on animal, untreatable human diseases can appear	0	−0.35	0^†^	0.47	0	−0.21
19	Vaccination reduce antibiotics use in livestock production	0	0.16	3^†^	1.71	0	0.03
20	*An antibiotic is always efficient on a bacterial infection*	*−1*	−0.71	*−3*	−1.68	*1*	0.84
21	To have the possibility to realize lab analysis will reduce antibiotics use	0	−0.13	3^†^	1.40	0	−0.16
22	Breeders always follow drug-seller advices	−1	−0.38	−1	−0.79	1^†^	0.86
23	We can trust in all drugs wholesaler supplier	−2	−1.03	−1	−0.72	1^†^	0.64
24	Antibiotics as preventive measure should not be use anymore	−2	−0.96	0^†^	0.28	−2	−1.26
25	*A better application of the law could reduce antibiotics use in livestock production*	*0*	−0.14	*0*	0.44	*−1*	−0.97
26	*It is better to use an antibiotic with a narrow spectrum than a large spectrum*	*0*	−0.25	*0*	0.32	*−2*	−1.03
27	**It is important to be formed about antibiotic's risks**	**2**	1.07	**3**	1.49	**2**	1.05
28	**If we misuse or overuse antibiotics, they can become inefficient on animals**	**1**	0.47	**1**	0.80	**1**	0.70
29	It is not a problem to give a less efficient antibiotic if it is less expensive for the breeder	−3^†^	−1.86	−2	−1.19	−2	−1.06
30	It is useless to inform breeders on the kind of antibiotic use and the reasons of their administration	−2	−0.82	−1	−0.77	0^†^	−0.23
31	**Everybody can sell drugs without having specific training**	**−3**	−1.73	**−3**	−1.82	**−3**	−2.04
32	When a product is efficient we have always to use the same	1^†^	0.64	−2	−1.07	−1	−0.54
33	Having only three or four different antibiotics is enough	0^†^	−0.37	−2	−1.19	−2	−1.14
34	Preventing diseases by vaccinate or cleaning the farms is less expense than using antibiotics	0^†^	0.29	2	1.13	3	1.53
35	*We must always give to the breeders what he wants*	*1*	0.55	*−3*	−1.91	*−3*	−1.37
36	*Antibiotics residues can be found in the soil or rivers*	*−1*	−0.51	*2*	0.80	*−2*	−1.12
37	**Using narrow spectrum antibiotics lead to inefficient antibiotics**	**−1**	−0.45	**−1**	−0.37	**−1**	−0.38
38	If more official controls were done, drug vendors will sell less antibiotics and win less money	−1	−0.70	−1	−0.32	−2	−1.01
39	**Conditions of storage of antibiotics are not important**	**−3**	−1.81	**−3**	−1.83	**−3**	−1.32
40	**If breeders take good care of their farm (cleaning, food, water, …) the antibiotics use will reduce**	**2**	1.11	**2**	0.80	**2**	1.05
41	Antibiotics should be change if the animals don't cure with the first treatment	2	1.42	1^†^	0.64	2	1.18
42	Prescription to buy drugs should become mandatory	0	−0.52	2^†^	0.82	0	−0.02
43	Information about withdrawal time must be given to breeders	3	1.52	3	1.70	1^†^	0.28
44	*More an antibiotic is expensive more efficient it is*	*−3*	−1.59	*−2*	−0.95	*1*	0.52
45	Antibiotic choice is independent of the specie to treat	−1	−0.64	−1	−0.65	0^†^	−0.07

† *Statement distinguishing factor from the rest*.

*In bold: consensus statements; in italic: distinguish statements*.

#### Consensus Statements

Six statements were consensus statements. For drug vendors, knowledge seemed to be important in AMU as they considered it mandatory to receive training to sell drugs (stat. 31: −3). Moreover, they thought that it is important to be informed about the risks of using AB (stat. 27: +2, +3). But it seems that they did not have a clear opinion about the source of resistance in livestock and particularly the impact of misuse or overuse of AB (stat. 28: +1). Also, they did not have a clear opinion on the way to use narrow or broad-spectrum AB and their impact on resistance (stat. 37: −1), revealing a knowledge gap regarding AB specificity. For them, conditions of storage were essential because the molecules that constitute the AB should be stored at a proper temperature and protected from the light stat. 39: −3). They said that despite the information given to farmers on storage conditions, these were not always complied with. As for breeders, they considered that keeping the farm clean was a good alternative to antibiotics (stat. 40: +1, +2) as this reduces disease transmission.

#### Discourse 2A “Trust in Antibiotics”

Six people shared this factor and represented 22% of total variance. This group was influenced by the “training” variable, with a majority lacking in training (*p* < 0.05). For these 3 technicians and 3 other salesmen, it seemed impossible to breed animals without antibiotics (stat. 9: −2). They felt that AB in prevention were essential to overcome stressful conditions like weather change or weaning (stat. 1: +2) and they did not see any link with this practice and manifestations of resistance (stat. 16: −2). They considered that technicians and veterinarians played an essential role for breeders. It was important to inform them about dosage and length of treatment (stat. 5: +2) and to write it down (stat. 11: +3) to be sure that they would complete the treatment even if animals were getting better. It was necessary to examine the animals before administering treatment (stat 2: +3) to choose the appropriate AB. In coherence with this group, 2B also believed that it was important to inform breeders of the kind of AB used, the reason (stat. 30: −2) and the withdrawal time (stat. 43: +3).

#### Discourse 2B “Belief in Alternatives”

This discourse was shared by 7 people, representing 20% of the total variance. The two veterinarians were part of this group, but no variables were significantly different. Contrary to the previous discourse, antibiotics were not essential in preventing disease (stat. 1: −2). They linked such practices with reduced effectiveness (stat. 20: −3). As such, and similarly to group 2C, people in this group believed it necessary to change AB (stat. 32: −2; stat. 33: −2) and to not always give the breeders what they want (stat. 35: −3). But, similarly to the others, they believed that prescriptions made by a veterinarian should be mandatory to buy AB (stat. 42: +2) and that it was necessary to raise awareness of breeders about the risks (stat. 15: −2). Moreover, they knew that AB residues can be found in the environment (stat. 36: +2). So, they were aware of the AMR problem and expressed the need to reduce AMU. They also supported alternatives: they were confident that vaccination could decrease AMU in the farm (stat. 19: +3, stat. 34: +2). Furthermore, they were the only group that wanted to carry out more laboratory analyses to reduce their AB consumption and to choose the right AB (sat. 21: +3), even though this was not yet achievable in practice.

#### Discourse 2C “Moderate Approach to Antibiotic Use”

Six drug vendors, representing 17% of total variance, belonged to this group which was heterogenous (no significant variable, Table 3). This group did not have a specific point of view regarding the topics investigated. The participants underlined the link between wholesalers and drug vendors. They stated they were satisfied with the number of drugs available from wholesalers (stat. 3: +3). Moreover, they agreed that some people sold fraudulent drugs that could be out-of-date or diluted (stat. 7: +2). We noticed that they preferred to use a broad-spectrum AB (stat. 26: −2). They did not believe there was any risk in using AB if one respected the utilization guidance (stat. 8: +3). They did not think that resistance could be transmitted between animals (stat. 10: −3) and that residues could be found in the environment (stat. 36: −2).

## Discussion

The aim of this study was to identify patterns of opinions, among breeders and drug vendors from Imerintsiatosika, regarding antibiotic usage. A semi-qualitative method, the Q methodology, was used to this end. The subjectivity of individuals was evaluated through face-to-face interviews in a preliminary approach to the problem of AMR and AMU in this province. Three groups of opinion were identified among both the breeder and the drug vendors populations, with high level of similarities between the two populations. In fact, even if they don't have the same statements, they were ultimately classified in three groups with similar perceptions. Indeed, although these two populations have different professions, they seem to have a comparable structure in terms of opinion. The group A had a positive opinion of AB and a low risk perception, contrary to group B that was aware of the risk of AMR and ready to rely more on alternative options. Group C was fuzzier, with less consistency in the discourse. *This could enable tailoring messages around AMU without multiplying their number too much although reaching different professionals*.

### Shared Opinions About AMU

Farmers and drug vendors were in favor of good management practices and biosecurity measures (stat. 30 for breeders and stat. 40 for drug vendors). Respondents considered that cleanliness, or good husbandry and management practices were factors of good health “*as living beings, humans or animals, we always need cleanliness. When we are clean, we are always in good health”* E8/R25. Protective measures to avoid people entering the farms were also commonly implemented by breeders (stat. 9), as stealing or poisoning pigs are common practices in Madagascar (personal source). But also because people were aware of the possibilities of pathogen transmission between humans and animals “*because diseases spread really fast, and we don't know from where a person comes from but if they enter the farm they can contaminate it”* E7/14, “*the person manipulates diseased pork meat and will come to my farm and touch the animals”* E10/R44. These biosecurity measures could be explained by the endemic presence of African Swine Fever in Madagascar. At the start of the epidemic, in 1996, half of the pig population of the country died (26). Meanwhile, breeders were afraid of disease transmission and reduced their movements between farms. As no national plan was implemented, nor resources allocated, these biosecurity measures were progressively reduced by breeders. However, farmers still keep in mind the danger of this epidemic and the benefit of biosecurity to control diseases. This represents an interesting finding to reduce AMU. Indeed, it has been shown that farms with a high level of biosecurity have fewer diseases (27) which lead to less treatment and that the evaluation of internal biosecurity (measures to reduce the spread of pathogens inside the herd) is inversely correlated to the use of AB for prophylaxis. which in turn leads to a reduction of antimicrobial use (28). As some people are already aware about the benefit of biosecurity, it will be easier to advocate the development of such measures to reduce AMU. Another common opinion among breeders was compliance with the withdrawal time (stat. 5) because “*we must wait for the drugs to be completely eliminated before slaughtering the animal otherwise it will transmit to disease to humans”* E15/R27. However, the national prevalence of antibiotic residues in pork carcasses was estimated at 28.3% in 2013 (29). The difference between our findings and this study can be explained by a number of reasons. It could be due to a misunderstanding of our statements. Indeed, qualitative data revealed that some breeders confused withdrawal time with treatment dosage. A lack of knowledge was also pointed out by a previous study in which 87% of farmers were not aware of the withdrawal times (12). Another explanation could be the gap between what farmers think should be done and what they actually do “*we suppose that the withdrawal time of the AB is 5 days and that the animal needs to be sold regardless, if breeders wait it will take 3 or 2 days, and they lose money”* E3/R1.

Breeders shared the opinion that they needed to ask for advice from drug vendors before buying AB (stat. 4). They were mainly interested in the AB dosage to avoid an “*overdose and kill the animal”* E26/R29 and because the veterinarian “*received training on the treatment of the animals”* E17/R31. However, direct observations, open-ended interviews and secondary data in Madagascar (12, 30) showed that self-medication was quite a common practice in breeders. Most of them considered that they had enough experience to avoid calling the veterinarian “*I called the vet when I started to breed, then I learnt the treatment that he was doing, so I don't call him now”* E15/R82. Moreover, self-medication was facilitated by easy access to drugs (sold in veterinary practices, drug depots and also in markets) and because a prescription was not always required. They also considered that the veterinarian was too expensive and too busy to come to the farm each time “*vets are lacking, no one will go to the countryside, to a remote place”* E15/R19.

### Differences in Discourses

In both study populations, two main points of view relating to AMU, AMR and alternatives were present. The main differences regarding AMU between groups A and B, in drug vendors and breeders, concerned the need to use AB in animal breeding (stat. 24, stat. 9), especially for prophylactic measures (stat. 32, stat. 1). These recommendations were provided by all kinds of drug vendors including those hired by companies which offer guidelines on good husbandry practices (vaccination programs, deworming, and prevention measures). “*We use it (AB) as soon as an animal is introduced in a farm, against stress, for example. Many breeders use vitamin AB in piglets during the first 3 days of their life because during this period they are sensitive to cold”* DS7/R43; “*I mix vitamins and AB in the pig's food when they are not yet sick and when they have diarrhea, I use another AB as a cure”* DS18/R25. “Anti-stress” medication was an AB and vitamin mix commonly used in Madagascar. The use of AB as a prophylactic measure was linked to the desire to increase productivity for most breeders “*because our vet advises us [to use AB as preventive measure] (…) [it] improves animal health (…) and I notice that it helps, and it is what led me to do systematic monthly treatments with the antiparasitic and the 20% [meaning Oxytetracycline 20%]”* E7/R15. This is approach was greater in imported chicken that were more productive but less resistant than *akoho gasy*, the local breed. However, people from group B did not believe that this was a good means of preventing disease because “*AB doesn't prevent the disease but cures it, it fights against the disease”* DS11/R18 and it could lead to their reduced efficiency “*if we give the AB while the animal is not sick, and we use the same when the disease breaks out, it will lead to AMR”* DS20/R31. One veterinarian belonging to group B said that this was outdated advice provided by some drug vendors. Attitudes toward alternatives differed between discourses. Vaccination was an interesting alternative for group B “*the first [alternative] will be the mandatory vaccination of the chicken, this is the most important, because if they are vaccinated, there will be less disease and so we will use less AB”* E9/R38 and because “*vaccination is for prevention, it is used before AB”* DS22/R6. In an European study, vaccination was perceived as the most feasible alternative to reduce AMU in 19 alternatives given to pig health experts, and the fifth in terms of perceived effectiveness which is consistent with our findings (31). However, to be considered as an effective alternative to AB in Madagascar, they need to be accessible by farmers. In pig and poultry production vaccines are not mandatory. So, the choice to vaccinate belongs to the farmers. But, this choice also depends on the access to vaccines, the presence of veterinarians and the skills of farmers. As it is in deficit in remote area, some projects provide training of vaccination with villagers (32).

People from group B also had a different perception of AMU risk than group A. Group B was aware of the AMR issue and its possible impact on human welfare. Moreover, respondents of this group did not use AB as a preventive tool, as they believed that AMU can be linked to resistance and they trusted alternatives. Whereas, group A did not notice any side effect of AMU, they had a positive opinion of AB and a low perception of risk. In this study, knowledge about AMR was related to lower AMU. So, we can hypothesize that raising awareness or knowledge around AMR will reduce AMU among the breeder and drug vendor populations. Similar results were found in previous studies looking at pig production in European countries where higher risk perception was related to lower AMU (5, 7, 33).

### Discussion on the Methodology

The Q methodology represents an interesting sociological approach to studying people's perceptions. Having a forced distribution helped people to prioritize their opinion and to identify the most important statements. The method is cheap, as little material is needed and it requires only a small number of participants (34); it is therefore well-adapted to low- and middle-income countries. However, the main limit of this method is that it is time-consuming for both participants and the research team (34).

Indeed, to be efficient, the Q methodology requires clear and comprehensible statements. It also calls for a maximum amount of information on the subject. To achieve these objectives, SSI and focus groups were run with different kinds of participants in order to collect a diversity of opinions to build the *concourse*. Moreover, two pilot studies were done to test our statements. If literature review enabled us to get design first interview guideline, PE and in particular SSI allowed us to modify some statements. For example, asking prescription and withdrawal time which are key for stakeholders in science and policy making but hard to understand by participants helped us to understand the local knowledge which demonstrate the usefulness of the method. This could not have been achieved solely by the literature review which is not country specific and may sometimes be outdated. However, as sometimes noticed during interviews, some participants still had trouble to understand certain statements. In particular, some words included in the statements that were hardly translated or exchangeable using a synonymous. For future studies, this problem could be limited by doing more SSI and pilot studies.

To guarantee the survey's objectivity, both investigators were trained in PE methodology and most of the participants (except the veterinarian from the city and one technician) were not aware of the survey's objectives. The data were also triangulated by open-ended interviews in order to check the right ranking of the respondents and to ensure a good comprehension of the statements. However, despite the triangulation, some erroneous ranking may have remained as coherence was only checked for a few statements (statements with extreme values). Furthermore, as the majority of respondents were poorly educated and sometimes illiterate, the duration of the interviews was generally longer than 1 h and half, leading to shortened open-end questions and potentially to a drop in respondents' concentration. Moreover, even if this bias was limited by the training of the interviewers, they can also influence the choice of the respondents in their decision-making process. Similarly, even if we followed a described methodology for the choice of the statements and the factors for the analysis, there is still a part of subjectivity as the final decision was done after a discussion within the research team. This subjectivity is inherent in qualitative research (35).

The main goal of the method was to maximize the diversity of opinions of the whole population (20). However, some farms were located in remote places and were difficult to access. Moreover, some of our respondents were included in the study because of their direct relationship with other participants, introducing some redundancies in the answer, as strongly related people can have the same opinion. In order to compensate for this lack and to obtain a wide array of perceptions, people were selected according to socio-demographic criteria.

It should be noted that this study was done in a restricted area and that, according to the Q Method, our respondents were not selected by random sampling (19). Despite this, the method does not allow a pattern of practices to be generalized to the entire country and no statistics regarding the number of people belonging to each group can be established. Nevertheless, it does form an interesting preliminary step to the development of further studies.

### Recommendations

AMU control seems weak in Madagascar (36) “*there is no control of milk, meat or eggs, there is not even a structure”* DS24/R4. Even though prescriptions are a legal requirement, over-the-counter sales are frequent, as is often the case in LMICs (37). This can lead to the misuse of AB with improper treatment and failure to comply with doses or withdrawal time. Another consequence of weak national regulations is the presence of an informal market. As for human medicines (38), drugs can be purchased from specific places that are well-known to the local population. No studies have been carried out on the informal veterinary market in Madagascar, although its existence was underlined by group C, among drug vendor population. This market leads to the availability of counterfeit, diluted or out-of-date drugs that can be a contributing factor to the development of resistance. It would be necessary to enforce regulations related to AMU and to monitor AMR (39). But the attitude of participants toward regulation is close to a neutral opinion, meaning that people in our study do not seem to be favorable toward regulations that aim to decrease AMR, or are not aware of them. Farmers and drug vendors must be involved in designing policy regulation to raise acceptance (7). This could be more easily achieved as these two groups have similarities in their opinion. The profession doesn't seem to have an important impact on the perception of individuals which can enable tailoring messages around AMU without multiplying their number too much.

Another issue is the low number of veterinarians in Madagascar. To compensate for this lack, technicians (with varying backgrounds) may provide treatment under the supervision of the veterinarian in charge of the area. In isolated places where there are no veterinarians or technicians, self-proclaimed professionals give advice and sells drugs without adequate knowledge (personal source). Selling drugs in these conditions could have consequences on AMU. Specific communication strategies around AMR should also target these populations.

Our study points to the fact that a proportion of the breeders and drug vendors interviewed were not aware of AMR and displayed excessive and improper AMU. When we compare the three groups, the variable education was significantly lower for group 1C, we may therefore hypothesize that the difficulty of forming an opinion was related to poor knowledge of AMU and AMR. In previous studies, knowledge seems to be correlated to a lower AMU (7) and greater awareness of AMR (40). As in our study, AMR awareness might be related to a more prudent use of AMU and to a greater confidence in alternatives. However, it could be possible that the socio-economic level of the respondents can be a confounding factor. Indeed, higher education can be related to a higher income (or the opposite) and better access to alternatives because they are more likely to leave near important cities. Nothing in the study or in the literature can help us to confirm this hypothesis. Breeders and drug vendors belonging to group A and C must therefore be informed about AMU risks. An increasing awareness of risk has already been underlined in many studies in Europe (2, 5, 33, 41). In Madagascar, this may be achieved thanks to “champions,” who are people with good AMU practices belonging to group B. People from the 2B groups could act as the promoters of good AMU practices by raising awareness of people around AMU guidelines and by explaining its possible impact on human health, as was proposed by one veterinarian of this group. Moreover, interviews seemed to indicate that there is a significant amount of communication among breeders. As the reduction of AMR requires a change in habits, group 1B farmers could act as “models,” using their farm to showcase other possible and efficient methods for farming animals. These breeders and drug vendors could also promote alternatives such as vaccination and biosecurity. People are already aware of certain good practices (e.g., biosecurity, withdrawal time) and this should help the message to be better understood and accepted. However, to develop this strategy is only possible if the access of alternatives is reachable (in term of cost and availability) to farmers.

## Conclusion

This study has underlined the need to educate breeders and drug vendors in Madagascar around better AMU practices and to promote awareness of AMR among the different stakeholders. By understanding the underlying factors that shape the perception of AB users, a more effective communication strategy could be developed to achieve accepted changes. A prudent use of AB and the development of alternatives could be advocated by “champions,” such as farmers and drug vendors, who show exemplary behavior. Raising awareness as to the public health risk of AMR could ultimately reduce AMU in the general population. However, future studies are required at the scale of the territory to generalize our findings.

## Data Availability Statement

The datasets generated for this study are available on request to the corresponding author.

## Ethics Statement

The study was approved by the Ethical Committee of Biomedical Research of Madagascar, with the authorization n° 037-MSANP/CERBM. Authorization of recording and written consent were obtained for each respondent before the interview.

## Author Contributions

CB, DK, and FG contributed of the conception and design of the study. CB and DR performed the data collection and analysis. DK, FG, AW, and LB reviewed the results. CB and FG wrote the first draft of the manuscript. DK, DR, AW, and LB reviewed the draft of the manuscript and wrote sections of it. All authors contributed to manuscript revision, read, and approved the submitted version.

## Conflict of Interest

The authors declare that the research was conducted in the absence of any commercial or financial relationships that could be construed as a potential conflict of interest.
