# Supplementary material for: Perception of Drug Vendors and Pig and Poultry Farmers of Imerintsiatosika, in Madagascar, Toward Risks Related to Antibiotic Usage: A Q-Method Approach
Source: Front Vet Sci. 2020 Aug 21;7:490. doi: 10.3389/fvets.2020.00490 (PMC7472779; doi:10.3389/fvets.2020.00490)
Supplement: Supplementary file 1 [file Table_1.DOCX]

Factor matrix and defining sorts after rotation: Q-sort factor loadings and defining sorts for breeders (a) and drug vendors (b)

| (a) |  |  |  |
| --- | --- | --- | --- |
|  |  |  |  |
| *n° Q-sort* | *F1* | *F2* | *F3* |
| 1 | 0.22 | -0.23 | 0.63 |
| 2 | 0.34 | 0.26 | 0.66 |
| 3 | 0.63 | 0.41 | 0.06 |
| 4 | 0.60 | 0.17 | 0.32 |
| 5 | 0.61 | 0.20 | 0.11 |
| 6 | 0.31 | 0.76 | 0.17 |
| 7 | 0.60 | 0.24 | 0.51 |
| 8 | 0.38 | 0.16 | 0.64 |
| 9 | 0.44 | 0.51 | 0.19 |
| 10 | 0.51 | 0.40 | 0.31 |
| 11 | 0.44 | 0.53 | 0.10 |
| 12 | 0.27 | 0.15 | 0.48 |
| 13 | 0.50 | -0.06 | 0.13 |
| 14 | 0.56 | 0.07 | 0.45 |
| 15 | -0.06 | 0.31 | 0.56 |
| 16 | 0.48 | 0.39 | -0.03 |
| 17 | 0.23 | 0.57 | 0.44 |
| 18 | 0.67 | 0.15 | 0.45 |
| 19 | -0.14 | 0.39 | 0.63 |
| 20 | 0.73 | 0.17 | 0.36 |
| 21 | *0.33* | *0.21* | *0.32* |
| 22 | *0.36* | *0.44* | *0.29* |
| 23 | 0.68 | 0.21 | 0.46 |
| 24 | 0.07 | 0.83 | 0.19 |
| 25 | *0.53* | *0.45* | *0.36* |
| 26 | 0.04 | 0.84 | 0.17 |
| 28 | 0.81 | 0.04 | 0.11 |
| 29 | 0.72 | 0.49 | -0.12 |
| 31 | 0.14 | 0.81 | 0.11 |

| (b) |  |  |  |
| --- | --- | --- | --- |
|  |  |  |  |
| *n° Q-sort* | *F1* | *F2* | *F3* |
| 1 | 0.47 | 0.32 | 0.58 |
| 2 | *0.55* | *0.50* | *0.26* |
| 3 | -0.09 | 0.20 | 0.56 |
| 5 | *0.47* | *0.36* | *0.59* |
| 6 | 0.65 | 0.19 | -0.02 |
| 7 | 0.78 | 0.13 | 0.27 |
| 8 | 0.65 | 0.04 | 0.38 |
| 9 | 0.39 | 0.27 | 0.56 |
| 10 | 0.77 | 0.30 | 0.27 |
| 11 | 0.20 | 0.60 | 0.28 |
| 12 | 0.16 | 0.32 | 0.58 |
| 13 | 0.32 | 0.55 | 0.30 |
| 14 | 0.74 | 0.27 | 0.06 |
| 15 | 0.00 | 0.90 | 0.04 |
| 16 | 0.45 | 0.05 | 0.61 |
| 17 | 0.25 | 0.65 | 0.43 |
| 18 | 0.66 | 0.39 | 0.24 |
| 19 | 0.14 | -0.03 | 0.81 |
| 20 | 0.22 | 0.72 | 0.21 |
| 21 | *0.44* | *0.25* | *0.50* |
| 22 | 0.41 | 0.67 | 0.17 |
| 23 | *0.42* | *0.39* | *0.43* |
| 24 | 0.27 | 0.76 | 0.05 |
