# Supplementary material for: Perception of Drug Vendors and Pig and Poultry Farmers of Imerintsiatosika, in Madagascar, Toward Risks Related to Antibiotic Usage: A Q-Method Approach
Source: Front Vet Sci. 2020 Aug 21;7:490. doi: 10.3389/fvets.2020.00490 (PMC7472779; doi:10.3389/fvets.2020.00490)
Supplement: Supplementary file 2 [file Table_2.DOCX]

General factor characteristics for breeders (a) and drug vendors (b)

|  | **Breeders (a)** | | | **Vendors (b)** | | |
| --- | --- | --- | --- | --- | --- | --- |
| *General factor characteristics* | *F1* | *F2* | *F3* | *F1* | *F2* | *F3* |
| Number of loadings Q-sort | 13 | 7 | 6 | 6 | 7 | 6 |
| Eigenvalue | 6.65 | 5.31 | 14.21 | 5.08 | 4.71 | 3.96 |
| % of explained variance | 22.94 | 18.33 | 14.21 | 22.09 | 20.47 | 17.22 |
| SE of factor scores | 0.14 | 0.19 | 0.20 | 0.20 | 0.19 | 0.20 |
| *Correlations* |  |  |  |  |  |  |
| *F1* | 1.00 |  |  | 1.00 |  |  |
| *F2* | 0.52 | 1.00 |  | 0.52 | 1.00 |  |
| *F3* | 0.59 | 0.48 | 1.00 | 0.58 | 0.42 | 1.00 |
