# Supplementary material for: Perception of Drug Vendors and Pig and Poultry Farmers of Imerintsiatosika, in Madagascar, Toward Risks Related to Antibiotic Usage: A Q-Method Approach
Source: Front Vet Sci. 2020 Aug 21;7:490. doi: 10.3389/fvets.2020.00490 (PMC7472779; doi:10.3389/fvets.2020.00490)
Supplement: Supplementary file 3 [file Table_3.docx]

Results of Kruskal Wallis test for socio-demographic variables between factors for breeders (a) and drug vendors (b)

| *Variables* | Gender | Age | Experience | Education | Status | Species | Type | Localization |
| --- | --- | --- | --- | --- | --- | --- | --- | --- |
| *P* value | 0.37 | 0.24 | 0.31 | 0.01* | 0.29 | 0.11 | 0.73 | 0.91 |

(a)

| *Variables* | Gender | Age | Experience | Job | Working with | Training |
| --- | --- | --- | --- | --- | --- | --- |
| *P* value | 0.92 | 0.68 | 0.21 | 0.54 | 0.70 | 0.052 |

(b)

* : p<0,05
